# Three Dimensional Honeycomb Patterned Fibrinogen Based Nanofibers Induce Substantial Osteogenic Response of Mesenchymal Stem Cells

**DOI:** 10.1038/s41598-017-15956-8

**Published:** 2017-11-21

**Authors:** Salima Nedjari, Firas Awaja, George Altankov

**Affiliations:** 1 Institute for Bioengineering of Catalonia (IBEC), Barcelona, Spain; 2 Biomedical Research Networking Center in Bioengineering, Biomaterials and Nanomedicine (CIBER-BBN), Zaragoza, Spain; 3 ICREA (Institucio Catalana de Recerca i Estudis Avançats), Barcelona, Spain; 4 Department of Orthopaedic Surgery, Medical University Innsbruck, Innrain 36 Innsbruck, Austria; 5 Regenerative Medicine Institute (REMEDI) and CÚRAM Centre for Research in Medical Devices, National University of Ireland, Galway, Ireland

## Abstract

Stem cells therapy offers a viable alternative for treatment of bone disorders to the conventional bone grafting. However clinical therapies are still hindered by the insufficient knowledge on the conditions that maximize stem cells differentiation. Hereby, we introduce a novel 3D honeycomb architecture scaffold that strongly support osteogenic differentiation of human adipose derived mesenchymal stem cells (ADMSCs). The scaffold is based on electrospun hybrid nanofibers consisting of poly (L-lactide ε-caprolactone) and fibrinogen (PLCL/FBG). Classical fibers orientations, random or aligned were also produced and studied for comparison. The overall morphology of ADMSC’s generally followed the nanofibers orientation and dimensionality developing regular focal adhesions and direction-dependent actin cytoskeleton bundles. However, there was an initial tendency for cells rounding on honeycomb scaffolds before ADMSCs formed a distinct bridging network. This specific cells organization appeared to have significant impact on the differentiation potential of ADMSCs towards osteogenic lineage, as indicated by the alkaline phosphatase production, calcium deposition and specific genes expression. Collectively, it was observed synergistic effect of nanofibers with honeycomb architecture on the behavior of ADMSCs entering osteogenic path of differentiation which outlines the potential benefits from insertion of such bioinspired geometrical cues within scaffolds for bone tissue engineering.

## Introduction

There is a sharp increase of bone disorders due to the aging of human population and relatively diminished physical activities. Bone tissue engineering (BTE) gained a considerable interest as it can provide an alternative therapy compared to the conventional treatment of patients using bone grafts^1^. Therapies that attract most interests recently are based on the use of mesenchymal stem cells previously committed, or not, towards osteogenic differentiation^2–4^. Adipose derived mesenchymal stem cells (ADMSC) draw particular attention, as these cells are readily available (including commercially) and possess clear, at least, three-lineage differentiation potential (osteogenic, chondrogenic and adipogenic)^5^. Moreover, they are abundantly expressed in adipose tissues (4% compared to 0.8% in the bone marrow)^5–9^ and provides less donor site morbidity upon harvest^10^.

*In vivo*, mesenchymal stem cells (MSC) behaviour depends on their three dimensional (3D) microenvironment in the niches^11^, in which they continuously exchange various chemical and physical cues with the extracellular matrix (ECM)^12^. Soluble factors and various spatially arranged ECM molecules (collagen, fibronectin, laminin, etc.) are strongly influential in MSCs behaviour^13^. Apart from the differentiation promoting effect of soluble factors, the role of the physical cues within the ECM are not well understood, particularly the role of fibrillary arranged matrix components and their 3D geometry^14^. *In vitro* studies utilizing photolithographic techniques clearly show that micro and nano-patterns strongly effect the differentiation of MSCs, toward osteogenic^15^, cardiogenic^16^ and neuronal^17^ lineages. For example Abagnale *et al*.^18^ showed that by varying the width of ridges and grooves, ADMSC differentiation can be switched to either adipogenic or osteogenic direction^18^. More recent studies of Lauria *et al*.^19^ showed that pillar patterns separation can also be used to control the calcium deposition by MSCs. These approaches however showed lack of efficiency in mimicking the real fibrillar organization of the ECM and its anatomically determined structure.

Nanofibers attracted significant recent interest as they can be made to mimic more closely the spatial organization of ECM^14,20^. There are different techniques to generate nanofibers, including phase separation^21^, self-assembly^22^ and electrospinning^20^. Electrospinning, however, provides distinct advantages as a well-established and versatile technique to produce differently organized fibrillary structures in nano- and micro-scale^14,20^. Indeed, the use of electrospun nanofibers has greatly improved the scope for preparing osteo-inductive scaffolds that resemble the fibrillary organization of the natural ECM^20^. Given that electrospinning provides opportunity to efficiently produce aligned nanofibers, the majority of the efforts have been concentrated on the role of aligned scaffolds in bone tissue engineering^23–25^. Uniaxially aligned PLGA nanofibers showed promise when are combined with hydroxyapatite for tuning its mechanical properties^24^. Modifying the casting parameters (rotation speed, input voltage, distance from the target, dimensions of the tip), the composition of the solution (type of polymer, solvent, concentration) or the overall electrospinning setup can results into differently organized fibrillar structures in terms of fibres diameter, composition and orientation^20,22^. However, comparative studies on the relative effects of nanofibers orientation (X - Y scale) and dimensionality (Z scale) are insufficiently addressed^26–28^, while any attempts for mimicking the complex tissue specific and evolutionary determined ECM architecture are rather missing.

Notably, the structure of natural bones is highly anisotropic^29^. The cortical bone is a solid structure with only a few small canals, while the trabecular bone inside is represented by a porous sponge-like scaffold rich in fibrillar collagen type I and resembling honeycomb structure^29,30^. Honeycomb structures are natural or man-made hexagonal assemblies, i.e. bee honeycomb, with the advantage of optimizing the amount of construction material to engineer stable and force balanced arrangements of compartments^31^. There were some previous attempts on using electrospun honeycomb structures as niches for osteoblasts differentiation^32^, however, these studies do not address the differentiation of stem cells^32^ though the implication of honeycomb structured ceramics to improve bon formation^33^ was explored. This paper concentrates on the specific behavior of mesenchymal stem cells on nanofibers architectured honeycomb scaffold, providing to our view naturally expired conditions that maximize their differentiation potential, an issue that was not explored in the past.

## Results and Discussion

In an attempt to mimic the naturally established bone structure, honeycomb arranged electrospun nanofibrous scaffolds were produced assuming it will provide optimal conditions for osteogenic differentiation of ADMSC’s. For that purpose we utilized a previously described electrospinning protocol^34^ yielding PLA/FBG nanofibers^34^ - containing the natural ECM protein fibrinogen (FBG). In this study however we used PLCL as core polymer (instead of PLA) as it was previously shown to have superior properties for the production of honeycomb structured scaffolds^32,35^. We added FBG to the system to make up a scaffold that provide cells with naturally recognisable ECM cue^36–38^. The multiple biological properties of FBG^39^ range from being essential for blood coagulation^40^ and wound healing^41–43^ to also acts as regular ECM in some tissues^44^. In addition, it is inherently easy to adapt FBG solutions for electrospinning^45^.

**Figure 1:**
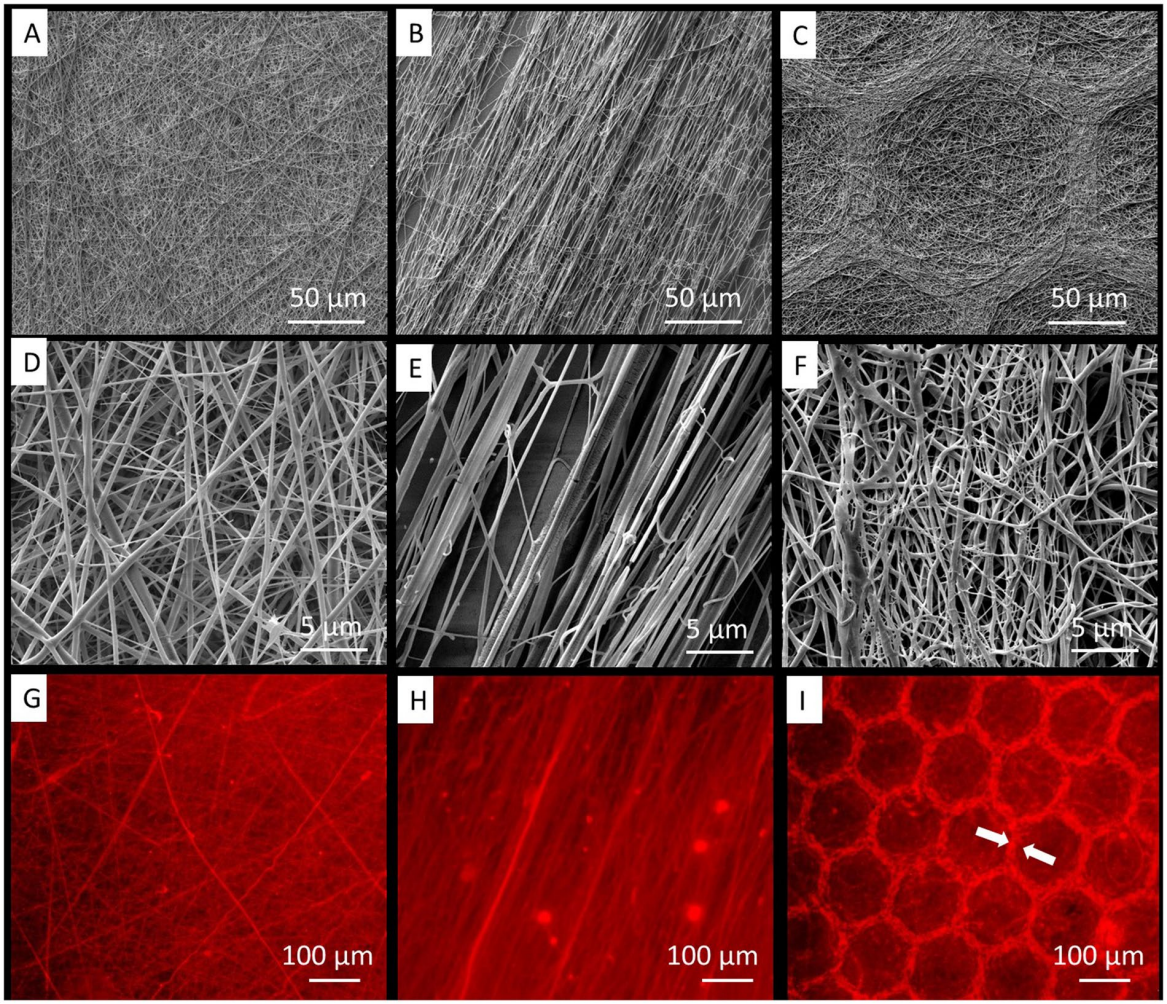
SEM images of random (**A**,**D**), aligned (**B**,**E**) and honeycomb shaped (**C**–**F**) PLCL-FBG nanofibers. The bottom row represents the immunofluorescent visualization of fibrinogen within the fibers (red) on random (**G**), aligned (**H**) and honeycomb (**I**) shaped scaffolds. The arrows on (**I**) indicate the higher accumulation of FBG at the walls of honeycomb shapes.

Though the electrospun FBG, alone, is well-recognized by the cells, it has poor mechanical properties, which hamper its biomedical application^36,37,45^. Adding PLCL to the system improves the mechanical properties of the scaffold while retaining the favorable cell recognition properties of the native FBG^32,34^. Our previous work showed that the optimal size of honeycombs that support osteoblasts interaction was 150–250 µm^35^, therefore we choose for this study an internal diameter of 160 µm. For comparison, we used random and aligned scaffolds, made in house based on a procedure that is described elsewhere^34,45^. Previous studies showed that by using honeycomb-shaped collector during electrospinning process, nanofibers preferentially deposited on the top of the patterns as the electrostatic field is higher there^32^. Conversely the random and aligned samples were deposited as flat layer. The overall topography of these scaffolds, as imaged by SEM at low (top row) and high (middle row) magnification is shown on Fig. 1.

Detailed image analysis shows that the fibres present bimodal distribution, in honeycomb scaffolds consisting of thin fibres with diameter of 195 (±50) nm, and thick ones of 462 (±117) nm. For the aligned samples it was 195 (±63) nm and 491 (±182) nm respectively, while for random ones it was 213 (±50) nm and 445 (±147) nm, respectively.

As concerned the honeycomb scaffolds, honeycombs walls were formed predominantly from thick fibres, while thinner ones formed the bottom of the honeycomb niches. We presume that during the electrospinning process, thicker portions of the jet, which are highly charged, are more “sensitive” to the electric field, therefore the fibers deposit preferentially on the top of the walls, thus choosing the shortest distance to the grounded electrode. Conversely, the thinner portions of the jet have higher freedom to deposit uniformly insight the niches. Nevertheless, the presence of thicker fibres at the edges contributes not only to the specific topography of the scaffold but also for its more stable mechanical properties.

The difference between the altitude at the top of the wall and the bottom (h) of the honeycomb was measured (using the specific SEM software) to be about 10 microns (±2 µm). The presence of FBG inside the fibres was confirmed by immunofluorescence (Fig. 1 inserts G, H, I). A higher amount of fibrinogen was detected on the walls of the honeycombs that obviously correlate with our observation that thicker fibres tend to deposit on the walls of the honeycomb scaffold.

In respect to the stability of the fibres, we previously showed that FBG is stably incorporated in the fibres in a distinct non-soluble form, which is not polymerized fibrin^36,37,45^. Interestingly, when extrahepatal FBG assembles as regular ECM (for some epithelial cells) it also forms non-covalent fibrils^44^, which presumably is the natural equivalent of the electrospun FBG^34,45^.

### Cellular interaction

**Figure 2:**
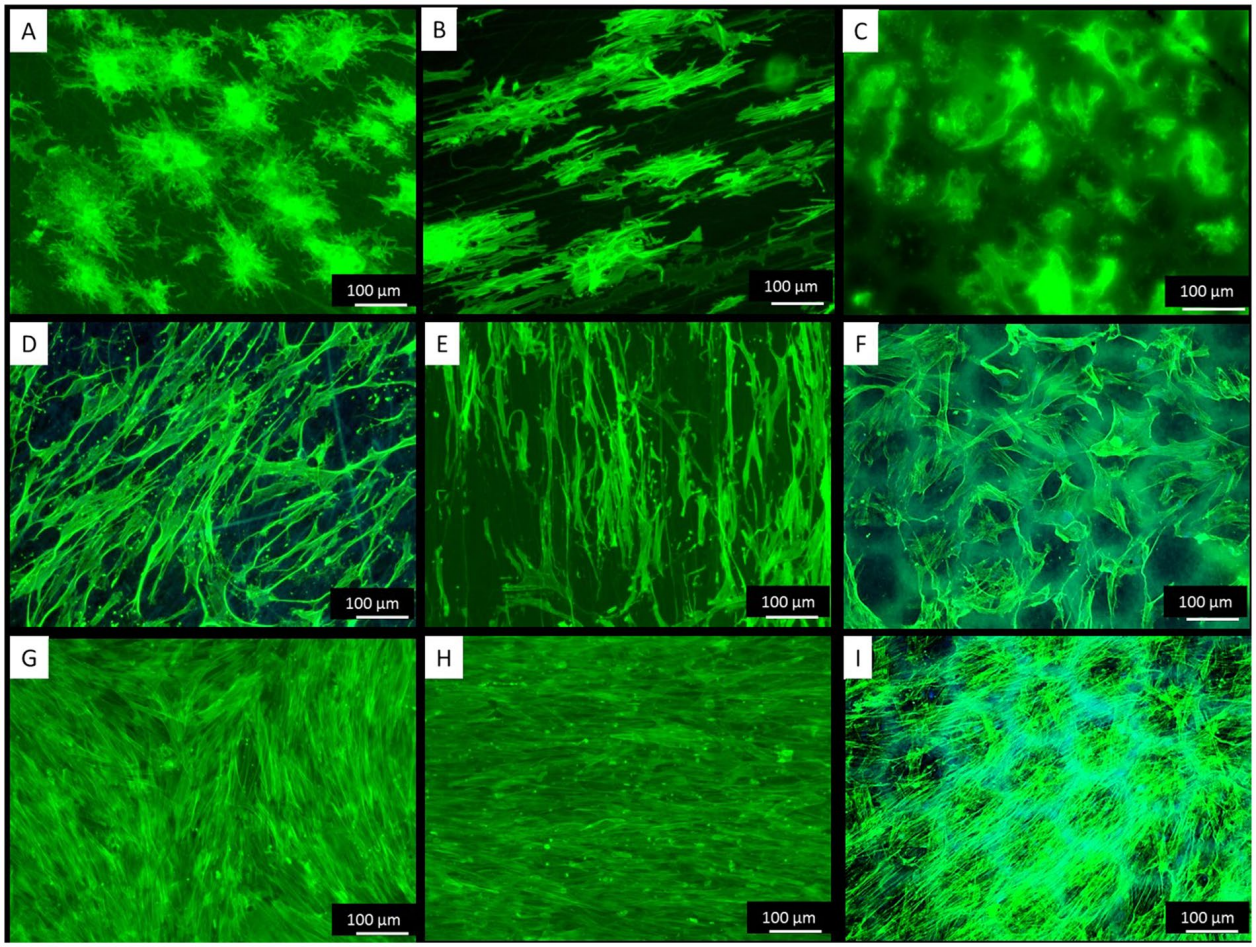
ADMSCs adhering on random (**A**,**D**,**G**), aligned (**B**,**E**, and **H**) and honeycomb (**C**,**F**,**I**) arranged nanofibers. After 5 hours of adhesion the cells are stained with Phalloidin (green) to view actin cytoskeleton at day 1 (**A**–**C**), day 2 (**D**–**F**) and day 6 (**G**–**I**) of incubation.

We deemed that morphological inspection of the cells is essential for their subsequent functional behaviour. As shown on Fig. 2, the overall ADMSCs shape was strongly affected by the substratum geometry. Оn random nanofibers the stem cells presented a stellate-like morphology (Fig. 2A) extending filopodia toward differently oriented fibers, a morphology better expressed on the next day of culture (Fig. 2D). Conversely, on aligned nanofibers, the ADMSCs showed a typically extended morphology (Fig. 2B) with stretched actin filaments aligning to the directions of nanofibers, a process also progressing at the next day of culture (Fig. 2E). Interestingly, on honeycomb shaped fibers, the cells initially concentrate inside the niches representing a rather rounded morphology characteristic for cells in 3D environment (Fig. 2C). The absent homogenous focus also indicates that the cells are located in different layers at the Z direction. These observations agrees with those made by Fukuhira *et al*.^46^ using another cells type showing that on honeycomb PLA films, chondrocytes reduced their points of attachments to the walls and adopted spherical shape inside the honeycomb.

On the second day of culture, however, the stem cells started to organize in network-like structures overlaying the honeycombs (Fig. 2F). Before forming networks, the cells presumably attached first to the top of the walls and then link each other over the honeycombs. This affinity of cells to attach top-hill seemingly is due to the accumulation of FBG there (Fig. 1I). At further incubation, the cells start to grow, and at the 6^th^ day they already cover all substrates, including the honeycombs (Fig. 2G–I).

**Figure 3:**
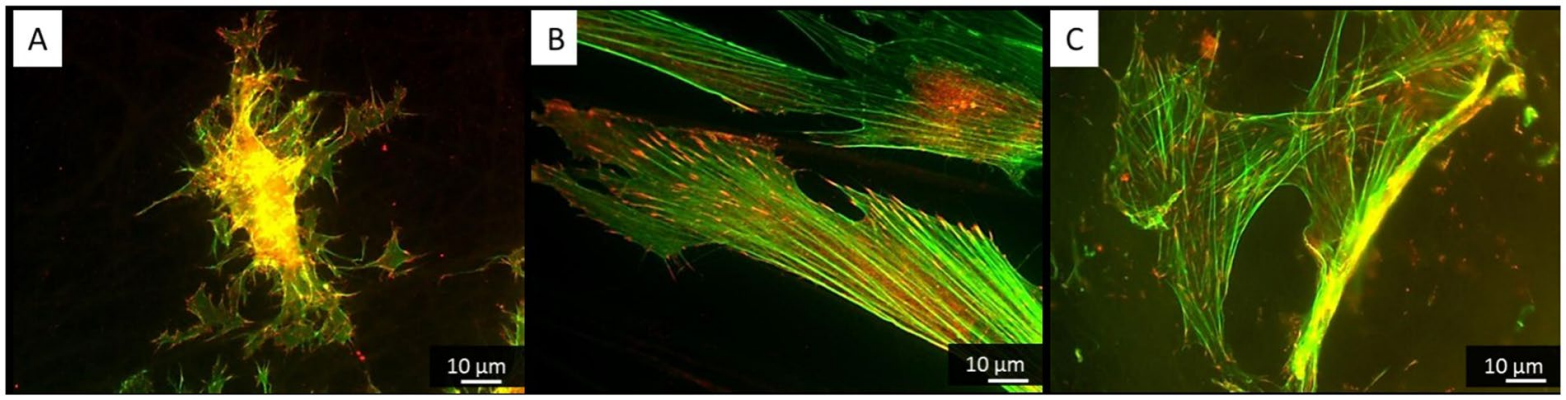
Visualization of actin cytoskeleton (green) and focal adhesions viewed (red) by vinculin of ADMSC adhering on random (**A**) aligned (**B**) on honeycomb (**C**) electrospun scaffolds after 5 hours of adhesion.

**Figure 4:**
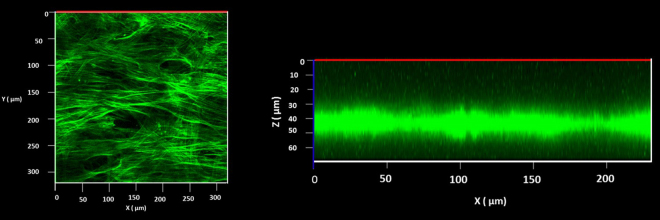
Confocal microscopy of ADMSCs grown on honeycomb scaffold. The cells are cultured for 6 days and stained for actin. The left image (**A**) represents the top view of the sample, while on (**B**) is shown the orthogonal view from the 3D reconstructed image of the cell layer.

On the other hand during their initial attachment the ADMSCs expressed well-developed focal adhesions complexes at all substrates (Fig. 3) suggesting that the PLCL/FBG nanofibers are generally friendly substratum and supports cellular interaction. On random and aligned nanofiber, however, the cells spread in a rather planar or 2D configuration, while on honeycombs the focal adhesions were observed both, at the bottom and at the top of the walls, suggesting that the confluent cells follow the shape of the honeycombs in the Z direction (3D). As the height of the wall is about 10 μm (i.e. comparable to the cells size), this means that they are initially constrained to follow the honeycomb shape. This was confirmed by the confocal images (orthogonal view) showing that ADMSCs layer has an irregular shape and presents periodic invaginations spaced in the range of 100 μm (Fig. 4).

Collectively, our morphological examination showed an initial tendency for rounding the cells on the honeycomb scaffolds, which is important as the initial shape of stem cells appears to play a substantial role in the initiation of osteochondral program^47,48^. Prior condensation, the stem cells having a typical mesenchymal morphology, when lose their substratum dependence, compensatory will increase their homotypic interaction^48,49^. Interestingly, similar tendency was observed also in our honeycomb environment, where during the initial attachment ADMSCs acquired a rounding shape, followed by a tendency to form networks at the next day suggesting an increase of intercellular adhesions - a behaviour not seen on all other samples. It seems honeycomb shape provokes specifically the homotypic cellular interaction presumably as an attempt to bridge the honeycomb curvature. Indeed, distinct studies highlights that stem cells need close contact in the niche to start differentiation^50^. Therefore we anticipate that the formation of cellular network on the honeycomb environment may also trigger the osteogenic programme of ADMSCs, which might be further augmented by the establishment of a near 3D environment on this substratum.

### Cell Proliferation

**Figure 5:**
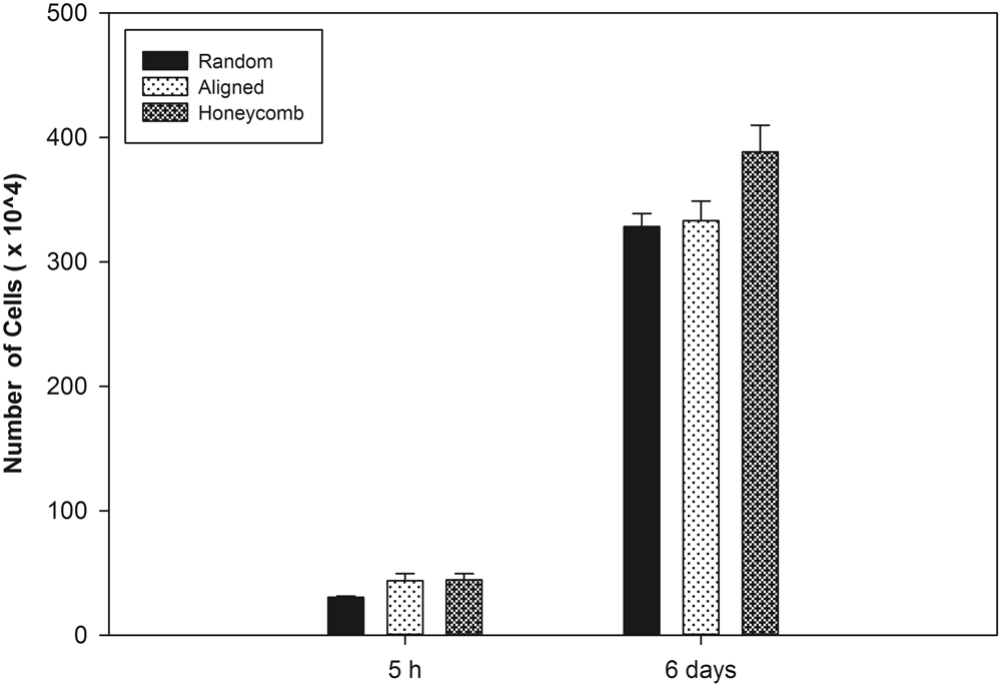
Cell proliferation on random, aligned and honeycomb electrospun shaped scaffolds as characterized by the significant increase of cell numbers at 6^th^ day of incubation versus their initial amount at the 5^th^ hour.

Cells number increased steadily over the first 5 days and at day 6 the ADMSCs were approximately 5 times as much, suggesting a substantial cell growth on all samples (Fig. 5). We could not distinguish however significant difference between samples; though some tendency for better cell growth is foreseen on honeycomb matrices. This confirms the conclusion that FBG/PCL fibers are generally friendly substrata for cells. Furthermore, it has to be mentioned that previous studies on cell proliferation using honeycomb configurations were rather contradictory. Some authors show that the honeycomb geometry itself do not disturb cell proliferation: for example, on honeycomb patterns obtained by casting of PLA with DOPE (surfactant producing honeycombs with diameter 5 µm and height 1 µm), fibroblasts attach and proliferate very well^51^. Moreover the cells produced more extracellular matrix that followed the fiber organization. Fukuhira *et al*.^46^ however show that the proliferation of chondrocytes is lower on honeycomb structured films, than on flat ones, but interestingly again secrete substantially more extracellular matrix when reside on the honeycombs.

In respect to the obtained equal growth of cells on random and aligned nanofibers, the data in the literature are also controversial. For example, Jahani *et al*.^52^ showed that though both aligned and random plasma treated PCL nanofibers are good substrates for rat MSC adhesion and proliferation, on random nanofibers they grow better^52^. Other studies however showed that the orientation of nanofibers do not have any effect on the proliferation of anchorage dependent cells, which corresponds well with the results obtained here and also in our previous studies^53,54^.

**Figure 6:**
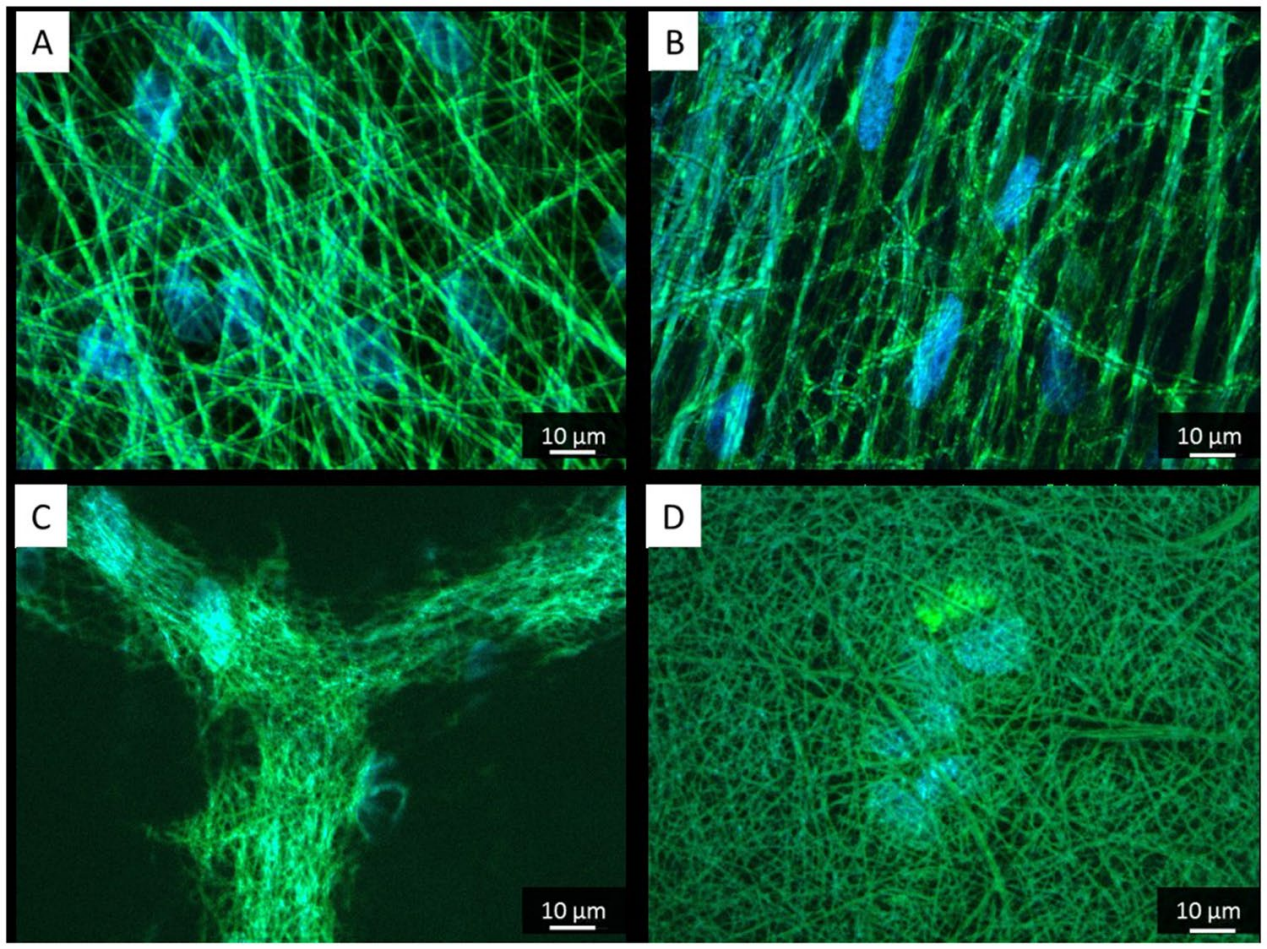
Fibronectin matrix secretion by ADMSCs after 6 days of culture on random (**A**) aligned (**B**) and honeycomb (**C**,**D**) arranged electrospun scaffolds. In (**C**) the objective is focused on the top of the walls while on (**D**) it is on the bottom of the honeycomb.

A sufficient production of ECM proteins is also an indicator for the efficiency of cellular interaction. Considering that ADMSCs secret significant amount of fibronectin (FN) *in vitro* ^34^, the deposition of FN matrix fibrils was used to functionally characterize the cells. As shown on Fig. 6 we observed significant FN secretion by ADMSCs which they deposit on the substratum following the fibers organization. Interestingly, the secreted FN assembled rather directly on the nanofibers than arranged in separate matrix fibrils as on plane substrata^55^. Presumably this is connected with the affinity of FN to bind FBG^43^ on the fibers surface, a binding demonstrated also on planar substrata *in vitro* with endothelial cells^55^.

On honeycomb scaffolds, although FN binds on both the top and the bottom of the walls (Fig. 6C,D), it predominantly assembles on the top of the so formed niches (Fig. 6C) presumably because more FBG accumulates there (Fig. 1I).

### Osteogenic differentiation of ADMSCs

**Figure 7:**
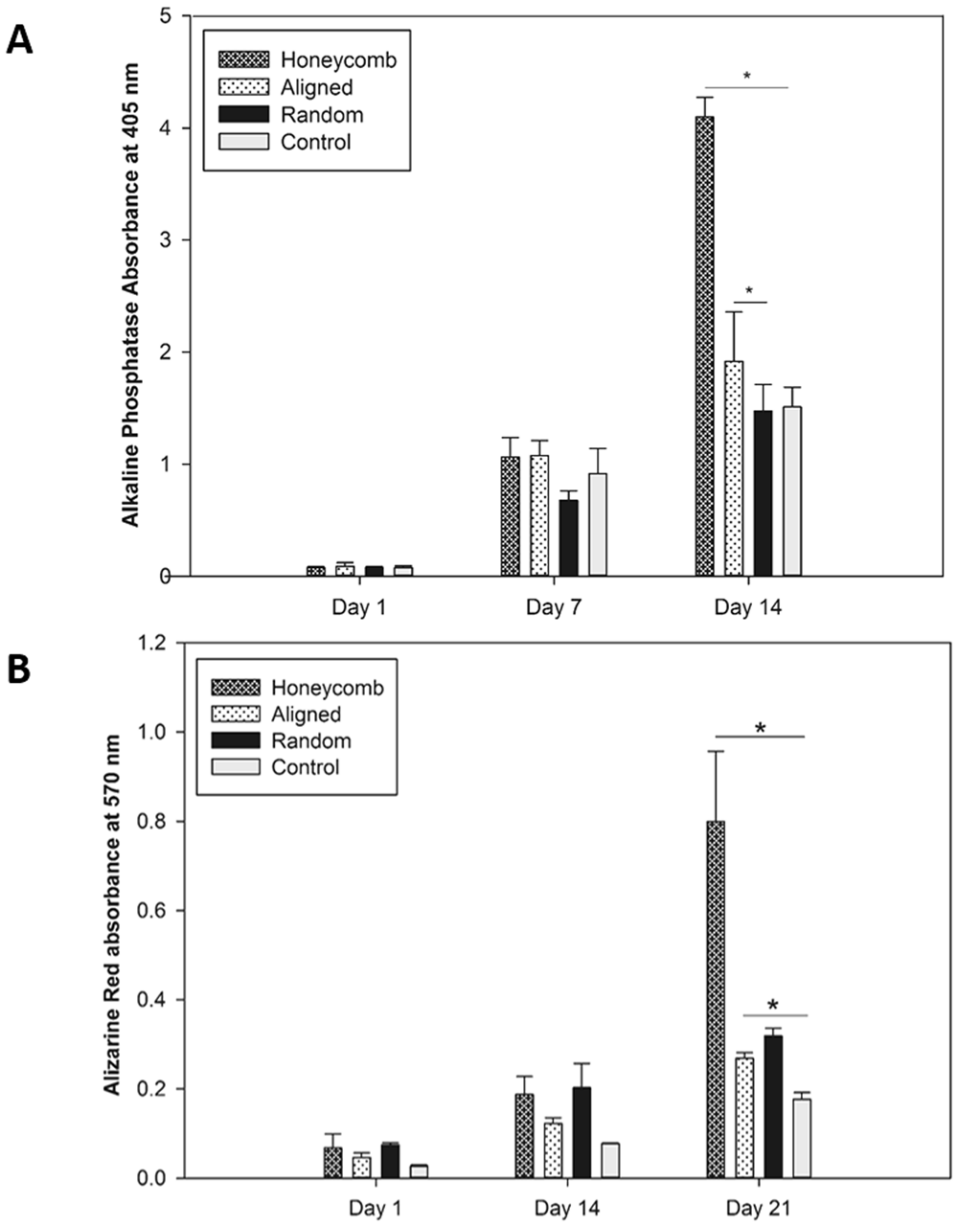
Panel A: Alkaline phosphatase activity of ADMSCs cultured for 1, 14 and 21 days on differently arranged PLCL/FBG scaffolds in osteogenic medium; Panel B: Alizarin red extraction assay of ADMSCs cultured for 1, 14 and 21 days on differently arranged scaffolds. As control was used FBG coated glass samples. Symbol * is added when difference is statistically significant (p < 0.05).

After the cells reached confluence (around day 5), the samples of each condition were switched to osteogenic differentiation medium for 7 and 14 days. As can be seen on Fig. 7A, ADMSCs on honeycomb configuration showed remarkable increase in alkaline phosphatase activity (p < 0.05), almost 3 times as much as on the control (FBG coated glass slides). On the other hand, having approximately same number of cells and same fiber constituents, stem cells on honeycomb structure expressed significantly higher phosphatase activity (p < 0.05) than on the other nanofibers configurations at day 14. This is a stark indication for the effect of honeycomb geometry on osteogenic differentiation.

The calcium deposition was also observed quantitatively via Alizarin red staining/extraction assay at day 1, 14 and 21 (Fig. 7B). A significant increase was observed at day 14 and 21 for all samples suggesting the generally supportive role of the nanofibrous environment for the osteogenic differentiation, confirming previous studies^1,22–24,26^. The highest Ca deposition however was again obtained in the honeycomb scaffolds (p < 0.05) compared to the other configurations, suggesting a strong impact of the honeycomb structuring on the mineralization process – an effect which was not described till now.

Mineralization was further studied by Von Kossa staining and the results are shown in Supplementary Information (Fig. S1) confirming that honeycomb scaffolds presented higher number of silver precipitates.

### Q-PCR data

**Figure 8:**
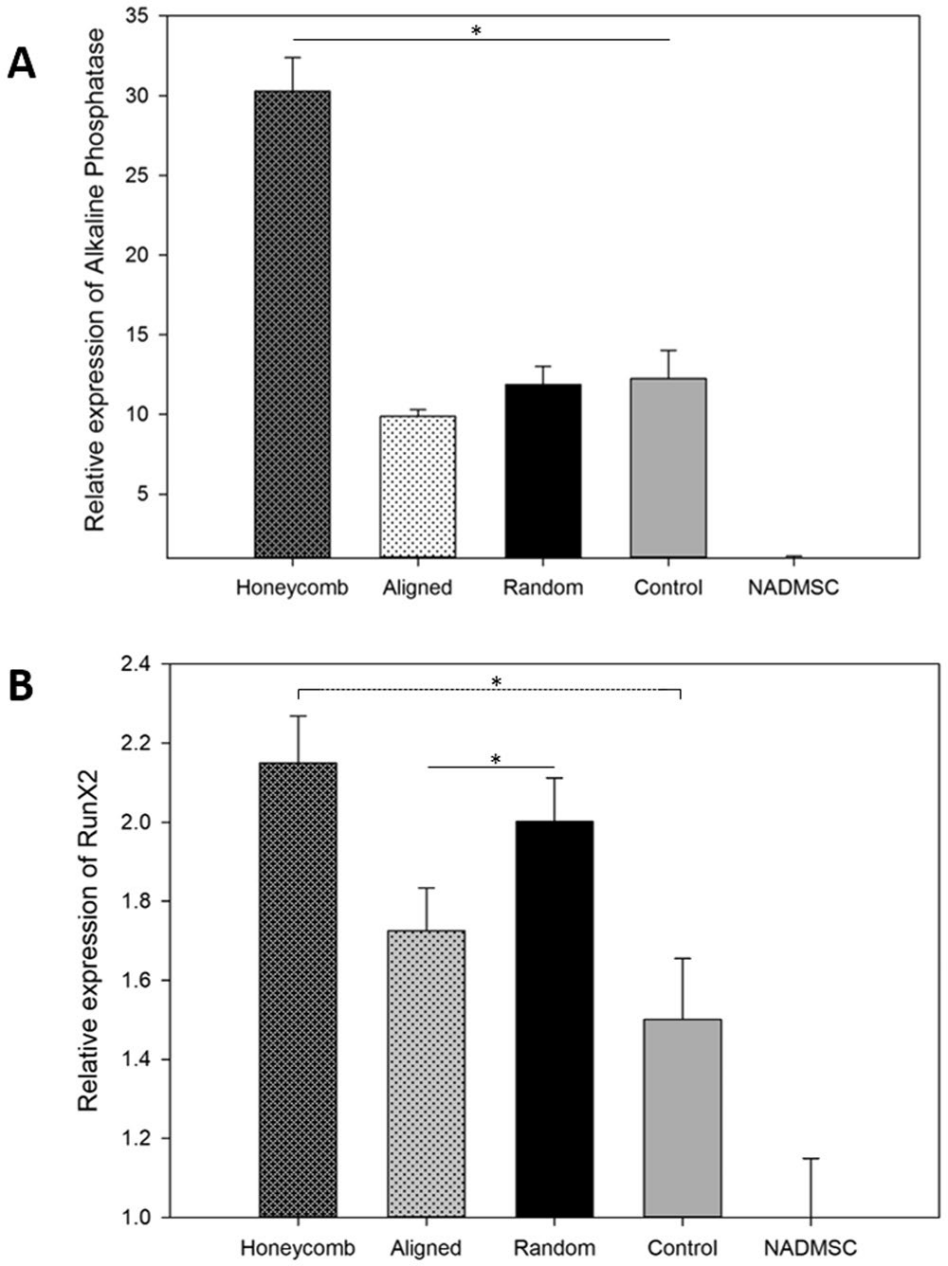
Relative expression of Alkaline Phosphatase gene (**A**) and RunX2 gene (**B**) of ADMSC cultured for 21 days in osteogenic medium onto differently arrange nanofibrous scaffolds. Symbol * is added when difference is statistically significant (p < 0.05).

The superior activity of ADMSCs on honeycomb scaffolds was confirmed by quantitative reverse transcriptase reaction for ALP and RUNX2 genes. As seen on Fig. 8A, the expression of ALP gene was again substantially higher on honeycomb scaffolds than on control and also than the basic activity of non-activated ADMSCs. In comparison to ALP activity measured enzymatically, the gene activity on random and aligned scaffolds was much lower than those observed earlier at day 14 with the direct measurements (Fig. 7A). This might be explained with the natural decline of the process at longer incubation^56^. Why it still persists on honeycomb scaffolds and decline on others at this stage remains unclear. The activity of RUNX2 gene generally followed the same trend: highest activity was observed for honeycomb scaffolds followed by insignificant decline on random (p > 0.05) and significant on aligned ones (p < 0.05).

The results above indicate that honeycomb organized PLCL/FBG nanofibers remarkably support the osteogenic response of ADMSC in comparison to “classically” arranged aligned and random scaffolds, confirmed quantitatively by the alkaline phosphatase activity, alizarin red extraction and real time qPCR. This raises an important query about the mechanism of this phenomena and its potential for application in bone disorders.

The important aspects of osteoblastic response^50^ within the hematopoietic stem cell niche has been particularly well studied^47^. Although the exact location and associated molecular signals of the MSCs niche remained elusive until recently^47,57^, in mammals stem cells are encased within the bone structure next to the endosteal bone, which means that the honeycomb structures within the cancellous bone might have an important geometrical impact on the bone regeneration. Studies on stem cells from diverse systems have shown that MSCs function is controlled by extracellular cues from the niche and by intrinsic genetic programs within the stem cell^33,47^ As for the observed tendency for homotypic interaction of ADMSCs on honeycomb structures, the embryonic “skeleton” also starts from the mesenchymal condensate, which is the future site of bone formation^58^, a process which involves the formation and remodelling of honeycomb niches. How the honeycomb features forms in the cancellous bone is not clear, but it presumably results from the concerted activity of osteoblast and osteoclasts remodelling the bone ECM^33^. Thus, the positive effect of a pre-patterned honeycomb scaffold on the osteogenic activity might be a complex fit-back response – i.e. the closely packed ADMSCs “see” an uncompleted bone-like structure and act to complete it. This correlates with the previous data of Kuboki *et al*. demonstrating that honeycomb shaped hydroxyapatite ceramics provoke bone formation, particularly those with large (300–400 μm) diameter^59^. No data however exploring the stimulating effect fibril-formed honeycomb architectures, which we are prone to explain as an additional effect caused by the fibrillary nature of bony ECM, where the role of the widely expressed collagen fibers is well documented.

Collectively, the overall aim for this study was to design a scaffold with bioinspired honeycomb arrangement based on using FBG-containing nanofibers with proven bioactivity. We also made a comparison with the classical nanofiber organizations, random and aligned, to eliminate the effect of the nanofibers environment itself. We employed a set of effective comparative evaluations of the initial cellular interaction and subsequent osteogenic differentiation of ADMSCs. Morphologically, a clear tendency for an initial cells rounding followed by ADFMSCs bridging in network was found on honeycomb scaffolds confirming that stem cells recognized the artificial honeycomb niches as 3D micro environment. This supports their homotypic interaction apart from aligned and random scaffolds, where cells are constrained to spread in a rather 2D environment. Honeycomb geometry substantially supports also the osteogenic differentiation of ADMSCs confirmed by superior cellular deposition of phosphate and calcium at different incubation period, and qPCR expression of the relevant gene marker of ALP activity.

The promotive effect of a fibrillary honeycomb scaffold on osteogenic differentiation has an important clinical meaning as may lead to the development of a novel bioinspired scaffold able to support the regeneration process through mimicking the conditions in the osteogenic stem cells niche.

## Methods

### Electrospinning

PLCL poly (L-lactide- ε-caprolactone) (70/30) (L-lactide/ε caprolactone) was purchased from Corbion Purac. Fibrinogen from bovine plasma and 1-1-1, 3-3-3-hexafluoroisopropanol (HFIP) was provided by Sigma Aldrich. PLCL was dissolved in HFIP at a concentration of 12% wt/V for 24 hours prior to electrospinning. Fibrinogen (FBG) was dissolved at 100 mg mL^−1^ in HFIP mixed (9:1) with 10× concentrated DMEM (Invitrogen). The obtained FBG solution was cleared by centrifugation at 4000 rpm for 10 minutes.

For the electrospinning, we used a tailor-made setup based on a high voltage supply (Glassman High Voltage Inc.), a syringe pump (Aitecs), and a grounded collector all fitted in a plastic chamber. PLCL and FBG were mixed in 1:1 ratio and loaded in a conventional 10 mL syringe (BD-Scientific). The pump flow rate was 0.6 mL h^−1^. The applied voltage was 20 kV, the distance between the needle tip and the collector was 13 cm.

### Preparation of honeycomb, random and aligned scaffolds

The honeycomb scaffolds were obtained using honeycomb shaped collector produced by photolithography on silicon wafers (MJB4, SÜSS Mask Aligner,). The photoresist used was SU-8 2050 (Microchem). A Univex 450B (Oerlikon Leybold Vaccum GmbH) electron beam evaporator was used to deposit a conductive layer (150-nm Au layer) on the collectors. The collector was with internal diameter of 160 µm. The width and the height of the honeycomb walls were 20 and 60 µm, respectively. After electrospinning the resulting membrane was peeled off from the collector and punched to obtain honeycomb samples with diameter of 14 mm.

Randomly deposited PLCL nanofibers were obtained by vertical electrospinning of the polymer solution onto glass coverslips (ϕ 14 mm, Thermo Scientific) placed on aluminum foil covering the collector. Aligned fibers were obtained using the same electrospinning conditions, but using an original method^60^ (see Fig. 1). Basically, two plastic discs (ϕ 120 mm) were mounted on a common axis separated at 100 mm. Thin metal wires were stretched between the discs peripheries to form parallel strings at 16 mm distance. While rotating the common axis (600–800 rpm), the nanofibers aligned between the metal wires were collected onto glass coverslips.

### Scanning electron microscopy

The morphology of nanofibers was observed by scanning electron microscopy (SEM) using (NOVANANOSEM 230, FEI) at a voltage of 5 kV. The height (*h)* of the fibrous honeycomb walls was measured by using the defocus/focus function of the scanning electron microscope, which exhibits a reported precision of 1 µm. The *Z* value provided as an estimate of the honeycomb height *h* was the average height calculated from 10 values.

### Cells

Human adipose derived mesenchymal stem cells (ADMSC) were obtained from Lonza Bio Whittaker (Verviers, Belgium). The cells were seeded in 75 cm^2^ flasks (Thermo Scientific, 136196) and expanded in DMEM/F12 (1:1) supplemented with 1% GlutaMAX™ (Gibco, 31331-028), 1% Antibiotic-Antimycotic solution (Gibco, 15240-062) supplemented with 10% Fetal Bovine Serum (FBS) (Gibco, 10270-106) in humidified CO_2_ incubator at 37 °C. The medium was replaced every 2 days. For the experiment, the cells were harvested at Passage 4 with Tripsin/EDT and seeded at density of 4 × 10^4^ cells/cm^2^ per sample (random, aligned and honeycomb arranged, with control fibrinogen-coated glass) placed in 24 well tissue culture plates (Thermo Fisher Nunc UpCell^TM^ Cat No 17489).

### Cell proliferation

Cell proliferation was assayed by direct counting of the cell nuclei viewed by fluorescent Hoechst staining. Image J software was used to count and analyze five randomly chosen images obtained from each sample at day 1 (5^th^ hour) and day 6 of proliferation.

### Overall cell morphology and visualization of focal adhesion complexes

The overall morphology of ADMSC cultured on random, aligned and honeycomb PLCL/FBG scaffolds was evaluated after 5 h, 1 day, and 6 days of incubation. To follow the focal adhesions formation and actin cytoskeleton reorganization, fixed samples were permeabilized with 0.5% Triton-X 100 (Sigma-Aldrich) and saturated with 1% BSA for 20 minutes to avoid unspecific binding before stained with a monoclonal anti-vinculin antibody (Sigma-Aldrich, V9264) (dilution 1:800) for 30 minutes at 37 °C. The samples were stained by goat anti-mouse IgG AlexaFluor 555-conjugated secondary antibody (Life Technologies, A11001) supplemented with Hoechst (dilution 1:500, Invitrogen) and FITC Phalloidin (Invitrogen 34580) for 30 minutes at 37 °C for simultaneous staining of the cell nuclei and actin cytoskeleton, respectively. At least three representative images were acquired for each sample at low (10X) magnification for overall morphology and at high (63X) magnification to view focal adhesions and cytoskeleton organization. To better evaluate the overall organization of cellular layers in random, aligned and honeycomb samples, confocal images were obtained on Zeiss confocal microscope where tangential reconstructions of the cell layers were done from a Z-stacks consisting of 10–15 confocal images using the corresponding software.

### Fibronectin matrix secretion

The deposition of fibronectin (FN) matrix was examined via immunofluorescence. For that purpose, ADSCs (4 × 10^4^) were seeded on honeycomb, aligned, random and control samples (triplets) and cultured for 6 days in basal medium containing 10% FBS. The samples were then fixed, permeabilized and stained with polyclonal anti FN antibody (Abcam, ab2413) followed by goat anti-rabbit AlexaFluor 448 IgG (Life Technologies, California, USA, A-11008) as secondary antibody. Dilutions used were: 1:500 for polyclonal anti FN antibody and 1:400 for Goat anti-Rabbit IgG, both incubated for 30 minutes at 37 °C. Finally, samples were mounted and viewed on fluorescent microscope Axio-Observer (Zeiss) as above.

### Induction of osteogenic differentiation

After 6 days, when the cell reached confluence, the medium was replaced with Osteogenic Induction Medium (OIM) consisting of DMEM supplemented with 100 nM dexamethasone, 50 μM l-ascorbate-2-phosphate and 10 mM β-glycerophosphate. The culture medium was replaced every two days. The osteogenic differentiation of ADMSCs was followed by quantification of alkaline phosphatase (ALP) activity and Ca deposition as explained below. In order to normalize the data for ALP and mineralization activity to cells numbers, the cell nuclei were counted in 3 separate samples from days 1 and 6. No significant differences were found (see Fig. 5) suggesting that after reaching confluence (for 6 days in growth medium) the cells stop growing and start to differentiate. Therefore the normalization to cell numbers was unnecessary in this case.

### Alkaline Phosphatase activity

ALP was determined at 1, 7 and 14 days in triplicate samples (for day 1 was considered the day when the osteogenic medium was added). The cells were washed and lysed with 200 μl of M-PER (Mammalian Protein Extraction Reagent, Pierce, 78501) per sample and after 5 min of shaking the lysates were centrifuged in Eppendorf centrifuge (14000 g for 7.5 minutes). The supernatants were transferred in a 96 well plate followed by addition of alkaline buffer (Sigma-Aldrich, A9226), distilled water (dH_2_O) and Phosphate substrate (Sigma-Aldrich, P5994) according the manufacturer protocol. The plate was finally incubated for 5 min at 37 °C in dark before 3 M NaOH was used to stop the reaction. The samples were measured at 405 nm using Microtiter plate reader. Standards prepared form p-nitrophenol (10 mM, Sigma-Aldrich, 7660) solutions in the concentration range between 6 × 10^−2^ and 1.0 mM were used to prepare a calibration curve.

### Mineralization

Mineralization was studied using Alizarin red staining. At day 1, 14 and 21, triplicate samples were fixed with 4% paraformaldehyde (10 min) and after washing twice with dH_2_O, 1 ml Alizarin red S solution (40 mM, pH 4.2, Sigma-Aldrich, A5533) was added to each well and incubated for 10 minutes at room temperature. The amount of matrix mineralization was quantified by staining the samples and dissolving the bound Alizarin red S with 10% Cetylpyridium chloride (Sigma, C0732) for 15 min at room temperature before measuring the absorption of each sample at 570 nm. Standards prepared form Alizarin S Red solutions in the concentration range 3.9 × 10^−2^ mM to 2,5 mM were used to produce a calibration curve.

For Von Kossa staining the cells were washed three times with PBs and fixed as above. Then 2.5% w/v silver nitrate solution was added to each sample and exposed to sunlight for 20 minutes. Then the samples were washed again with milli Q water and the unreacted silver was removed with 5% of sodium thiosulfate for 5 minutes.

### Real time PCR

Total cellular RNA was extracted from the middle tissue discs by using RNeasy Mini Kit (QIAGEN, Germany). Reverse transcription was performed by processing of 10 µL of three times diluted RNA-eluate with High Capacity cDNA Reverse Transcription Kit (Applied Biosystems) according to the suppliers protocol. Quantitative PCR analysis of the gene expression was carried out with fast real-time PCR System (Applied Biosystems 7900HT). Briefly, a single qPCR reaction consisted of mixing 10 µL Taqman Gene Expression Master Mix (Applied Biosystems), 1 µL TaqMan primers (Applied Biosystems), 5 µL nuclease-free H2O and 4 µL of sample cDNA. Some details related to TaqMan primers used for the detection of Alkaline Phosphatase gene (Primer: Hs01029144_m1***)***, RUNX2 gene (Primer: Hs01047973_m1) and GAPDH (Primer: Hs02758991_g1***)*** as housekeeping gene. Each sample was run in triplicate and the cycling program was set on 95 °C for 10 min, followed by 40 cycles of 95 °C for 15 s, and 60 °C for 1 min. Data analysis was performed by DataAssist Software (Thermo Fisher Scientific, Spain). Comparative CT analysis was used to determine the relative expression of each target gene. The amount of the target gene was normalized to GAPDH as housekeeping gene. Transcription levels were expressed as fold change from the calibrator control value. Non-stimulated confluent ADMSC were used as a control in all qPCR experiments.

### Statistical analysis

Data from all quantitative analysis were expressed as mean ± standard deviation and subjected to one-way ANOVA variance analysis. Each experiment was performed in triplicates and repeated at least two times. Statistical significance was determined by Student’s t-test. Probability values with p < 0.05 were considered as statistically significant.

## Electronic supplementary material

Supplementary Information
