# Supplementary material for: Three Dimensional Honeycomb Patterned Fibrinogen Based Nanofibers Induce Substantial Osteogenic Response of Mesenchymal Stem Cells
Source: Sci Rep. 2017 Nov 21;7:15947. doi: 10.1038/s41598-017-15956-8 (PMC5698442; doi:10.1038/s41598-017-15956-8)
Supplement: Supplementary file 1 — Supplementary Information [file 41598_2017_15956_MOESM1_ESM.doc]

Three Dimensional Honeycomb Patterned Fibrinogen Based Nanofibers Induce Substantial Osteogenic Response Of Mesenchymal Stem Cells

Salima Nedjari^1^, Firas Awaja^1^, George Altankov^1, 2, 3^*

**Supplementary Information**


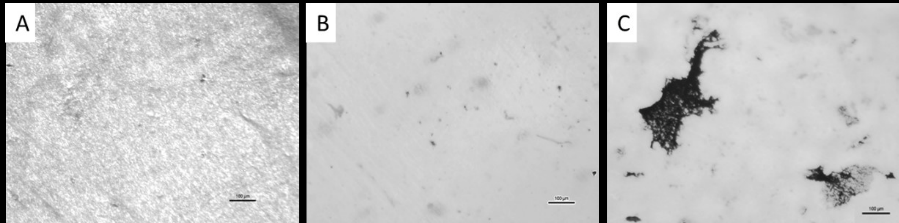


**Figure S1:** Von Kossa staining of ADMSCs cultured for 21 days on (A) random (B) aligned and (C) honeycomb arranged PLCL-FB scaffolds.

Figure S1 shows the Von Kossa staining observing the precipitation reaction when silver ions react with phosphate (not calcium). As shown on S1, the silver precipitates (arrows) at day 21 were substantially larger on honeycomb samples (Fig S1-C) compared to the other fibrous scaffolds where only point- precipitations were detected.
